# Psychosocial issues discovered through reflective group dialogue between medical students

**DOI:** 10.1186/s12909-017-1114-x

**Published:** 2018-01-10

**Authors:** Shao-Yin Chu, Chi-Wei Lin, Meei-Ju Lin, Chin-Chen Wen

**Affiliations:** 1 Department of Pediatrics, Buddhist Tzu Chi General Hospital, Hualien, Taiwan; 2 Department of Medicine, College of Medicine, Tzu Chi University, Hualien, Taiwan; 3 Department of Counseling and Clinical Psychology, National Dong Hwa University, Hualien, Taiwan; 4 Department of Human Development and Psychology, Tzu Chi University, Hualien, Taiwan; 5 Department of Counseling and Clinical Psychology, National Dong Hwa University, Hualien, 97401, Taiwan, Republic of China

**Keywords:** Medical student, Psychosocial issues, Reflective group dialogue, Thematic analysis, Biopsychosocial model

## Abstract

**Background:**

The biopsychosocial model is a comprehensive approach emphasizing holistic medical care. However, medical curricula that incorporate narrative reflective writing and group dynamic discussion of psychosocial issues among patients and their family members in reflective dialogue groups are currently underutilized. The aim of this study was to determine psychosocial issues among patients and their family members through medical students’ reflective dialogue groups.

**Methods:**

This study was completed as part of a pediatric clerkship. Fifty medical students were rotated to the department of Pediatrics. They completed a narrative writing assignment concerning patients’ psychosocial issues and participated in a reflective group discussion during the rotation. The recordings of the six reflective group sessions were transcribed for thematic analysis. A six-step theme generation process was conducted in the first reading stage of all transcripts by four researchers. Subsequently, initial codes were generated and potential themes sought before possible themes were reviewed and thematic maps generated. Names for each theme were defined and a scholarly report of the analysis was presented through a consensus-based approach.

**Results:**

A total of 108 psychosocial issues were coded and categorized as the following six main themes: medical communication, the intricate medical ecological system, role and function of a family, development of medical professionalism, ethical dilemmas, and various patient perspectives from diverse cultural backgrounds. All these themes underlie the complexity of clinical encounters.

**Conclusions:**

Clinical care is an extremely complex interactive ecological network involving human behavior, family, and public health care systems. The discovery of psychosocial problems by medical students as narrators in this study illustrates that medical care should focus not only on illnesses but also patients’ psychosocial narratives.

## Background

Building on the biopsychosocial (BPS) model, this study used thematic analysis to identify psychosocial issues disclosed by medical students during a small group discussion. While the biomedical approach adopts a reductionist view that mainly focuses on disease, the BPS model considers not only the importance of biomedical factors but also psychological and social factors, both of which significantly influence human disease [1–3]. The BPS model is clinically oriented, teachable, and reinforced in clinical medical education. The model represents a method for understanding how degrees of suffering, disease, and illness are affected by multiple levels of natural systems from the societal to the molecular level [2]. At the practical level, the BPS model emphasizes the importance of understanding patients’ subjective experiences and life stories as an essential contributor to determining appropriate clinical practices. Following this model, physicians are responsible for not only arriving at accurate biological diagnoses but also for learning to comprehend illnesses and health from patients’ perspectives by providing patients with opportunities to address their concerns as a means of understanding their expectations and issuing health-related information. This relationship-centered model guides clinical conditions toward a dialogic model that enables a more holistic and humanistic response [3].

In accordance with the BPS model, the emerging narrative in medicine calls for physicians to practice medicine with greater skill, empathy, reflection, professionalism, and trustworthiness [4], asserting that biomedical competent practices alone are insufficient to help patients grapple with deteriorating health or find meaning in their suffering [1]. In addition to objective scientific knowledge, the narrative asserts that medically informed physicians must develop competency in acknowledging, absorbing and interpreting, as well as understanding, stories and hardships narrated by patients, accepting their meanings and acting on the patient’s behalf [4, 5].

Given that reflective writing represents a valuable approach to developing narrative competency, medical students can benefit greatly from reflecting on and recounting their encounters with patients and family members [6–8]. Because forming narratives in the context of their illnesses is a highly complex task for patients and their family members, we attempted to outline this context and focused on biopsychological and social concerns to guide students to present their narratives in a structured and reflective manner (i.e., reporting, responding, relating, reasoning, and reconstructing) [9, 10]. The medical students in this study were encouraged to listen to patients’ stories to form biological diagnoses and develop psychosocial perspectives on specific problems that were observed and addressed.

The BPS model is fundamentally rooted in systems theory, which views nature as a hierarchically arranged continuum where the more complex, larger units are superordinate to the less complex, smaller units; notably, the individual (person) simultaneously stands at the highest level of the organism hierarchy and is the lowest unit of the social hierarchy [1]. Engel indicated that “Each system implies qualities and relationships distinctive for that level of organization, and each requires criteria for study and explanation unique for that level” [1]. The present paper suggests that health professionals develop a systems theory perspective and learn to assess intercorrelated problems.

Within the systematic BPS model and medical narratives, psychosocial issues are mainly components in the social hierarchy that comprise personal, interpersonal, familiar, communal, societal, and cultural levels. Such issues are relevant and indispensable elements in medical diagnoses and treatments, and thus should be included in medical education. Psychosocial issues are events or disruptions in a person’s life such as housing concerns, financial matters, domestic abuse, grief and loss, isolation, and other factors specifically related to mental health, depression, anxiety, and adjustment. In addition, psychosocial issues can be defined as conditions or problems in personal, social, occupational, and environmental domains, as well as those related to physical and mental health [11, 12]. A lack of understanding and sensitivity toward psychosocial issues can influence patient responses to interventions; thus such issues should not be overlooked [13]. By addressing psychosocial issues in a reflective dialogue group, the students in this study were able to collectively learn about their peers’ perspectives and the scope of severity of such problems, thereby enabling them to act on their insights. Moreover, discerning psychosocial issues among student reflections can sensitize medical teachers toward these themes and enable them to engage the students in an in-depth discussion. Most universal psychosocial issues are culturally-bound to a certain degree. Familiarity with common or specific challenges is crucial for physicians and health professionals to provide appropriate subjective care for each patient.

It is advantageous to educate students to respect and recognize essential psychosocial issues and diseases in patients through reflective practice. By facilitating a reflective dialogue group (which formed a platform for narrative and feedback), this study discovered and identified psychosocial issues among patients and their families from medical students’ reflections and narratives, as well as feedback from peers and mentors in the group process. In addition, this study identified the themes of psychosocial issues found among patients and their families from medical students’ reflections and narratives.

## Methods

### Course design and learning objectives

A narrative writing project was conducted among fifth-year medical students during their clerkship. The following learning objectives were designed: 1) to listen to and interact with patients and their family members to gain clinical exposure not only to their diseases but also their life stories and concerns; 2) to observe and identify psychosocial issues among patients and their family members; and 3) to perform self-reflection regarding self and others through narrative writing using Gibb’s reflective model [14]. The writing was structured around reporting identified psychosocial issues, the initial response of the student, the patient, and the patient’s family, the comparison of past experiences, reasons why the psychosocial issues occurred, and finally reconstructing how to make an action plan to solve the issues; and 4) to debrief as a re-interpreter during small group discussion.

### Group discussion

A total of 50 students rotated to the pediatric department for one month (7–9 on each rotation). All of them consented to participate in the study, and they all had the opportunity to opt out of the study (The study was also approved by the IRB). Each student completed the reflective writing assignment and participated in one of the six group discussion sessions. Each group discussion lasted for two hours. During the group discussion, the students debriefed the psychosocial issues of the patients and their family members. They described the differences relating to their past experiences in how they responded to patients, and how subsequent variations impacted themselves and their patients. In addition, the role of a medical student facing such psychosocial issues was addressed. Finally, a practical action plan was proposed. After the debriefing process, each student received feedback from one peer and one teacher before an open discussion was conducted among all group members.

### Transcripts

The medical students’ reflective group dialogue was recorded and permission was given for transcripts to be typed, of which six copies were made.

### Thematic analysis

The recordings of the six reflective group sessions were transcribed for thematic analysis. Four authors independently reviewed the materials and jointly conducted a six-step theme generation process. First, a general reading was conducted to gain an overall understanding of the narratives and code the text into independent preliminary themes. Subsequently, a process of peer debriefing, reviewing, defining, naming, and discussing was performed by the four authors until consensus on the final themes was reached to ensure coherence and consistency [15].

Ethical approval was obtained from the institutional review board at Buddhist Tzu Chi General Hospital (IRB101–80). Informed consent was received from all participating medical students.

## Results

Six discussion groups were recorded over a one-year period. A thematic analysis approach determined six major themes, which were presented in a three-layer format representing coded components grouped into subthemes and categorized into main themes.

### Theme 1: Medical communication

**Fig. 1:**
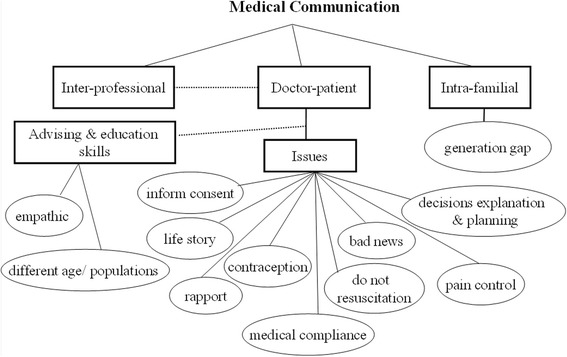
Conceptual map of medical-communication-related psychosocial issues

See Fig. 1. Interpersonal communications occur between physicians and patients, members of various professions, and among intrafamilial members. In medical contexts, communication challenges include the need for physicians to build rapport with patients and family members, listen to their life stories, gain informed consent from patients, communicate daily information about disease progression and new decisions, explain problems regarding resuscitation and the concept of contraception among the young adolescence population, deliver bad news, explain how to manage pain, and explain the importance of drug compliance. In addition, advising and educating patients are crucial elements that require empathic listening and special communication skills, particularly for pediatric patients of various developmental ages and special populations, such as patients with attention deficit hyperactivity disorder or malignancy. Inter-professional consultations in clinical settings remained a focus of treatment throughout this study. Ongoing discussion of such concerns enables medical students to learn from and collaborate with various types of health professionals.

### Theme 2: Diverse patient perspectives

**Fig. 2:**
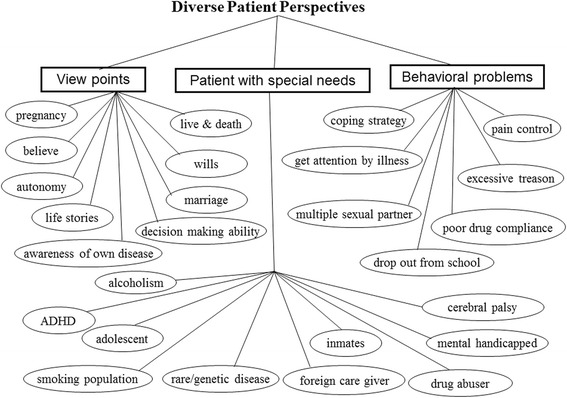
Conceptual map of patient-perspective-related psychosocial issues

See Fig. 2. A wide range of patient perspectives emerged during this study. Challenges related to poor drug compliance were easily identifiable among the pediatric population. Patients with special needs, such as children with lower intelligence and those with rare genetic diseases, also have distinct concerns that must be addressed. Behavioral problems, such as multiple sexual partners (potentially resulting in underage pregnancy), adolescent rebellion that affects medical treatment, emotional instability, and a lack of awareness of one’s disease status or self-image all engender a need for extra attention throughout the course of an illness. Emotional responses were unique in each patient as well; for example, those related to pain tolerance and coping strategies for a single clinical decision could influence adherence to medical care and drug compliance. The students also acquired firsthand knowledge about popular folk beliefs, such as the belief that pregnant women should not drive a nail in the house throughout the baby’s gestation, because the fetus will be at risk for a dangerous condition (such as having a cleft lip and/or palate). During the reflective dialogues, patients with special needs were identified as a group more prone to self-harm than others. Finally, through the group discussion, the students were able to understand how cultural background contributes to behavioral problems among patients and their family members during medical treatment. For example, in rural or traditional communities, especially among people with poor health literacy, faith in folk prescriptions or herbal medicine greatly affects drug compliance.

### Theme 3: Family

**Fig. 3:**
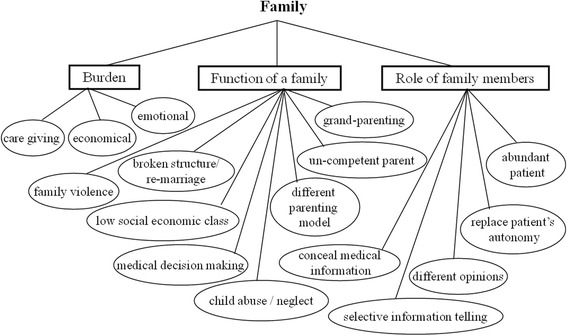
Conceptual map of family-related psychosocial issues

See Fig. 3. Diverse family backgrounds, regarding ethnicity, socioeconomic status, and educational status, were concretely identified and fluctuating functionality in families was observed. Contributing to medical decision-making is a primary indicator of a family’s functionality, but high risk family scenarios (e.g., violence at home, incompetent parenting, child abuse, neglect, and abandonment) were frequently identified. Most patients observed by the medical students in this study had little understanding of their diseases and because they were children, they may not have been able to indicate willingness to be involved in medical decision-making. By contrast, some patients’ family members were instrumental in such decision-making, which could often compromise patient autonomy. Disputes among family members render treatment decisions extremely difficult for physicians. In addition, unfavorable phenomena were observed, such as real clinical information being concealed or facts being selectively provided. Finally, caregiving was identified as an economic and emotional burden on patients’ family members, and as being mostly performed by women.

### Theme 4: Ethical dilemmas

**Fig. 4:**
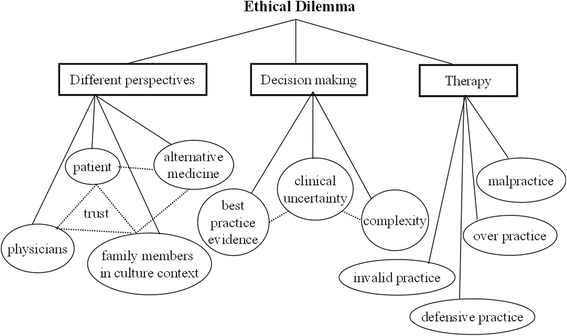
Conceptual map of ethical-dilemma-related psychosocial issues

See Fig. 4. Several topics related to ethical dilemmas were discovered in the medical students’ group reflective dialogue transcripts. The ethical dilemmas were observed to have originated from the various perspectives, expectations, beliefs, thoughts, and coping strategies of patients and their family members; the trust or lack thereof between medical personnel; and social and cultural traditions and norms. For example, lumbar puncture procedures in children are usually refused by grandparents due to their traditional belief that such procedures will disrupt the water of the dragon’s bone, and that tapping it will bring disaster. In addition, ethical dilemmas are affected by clinical uncertainty, the complexity of a disease and its unpredictable progression, and the applicability of the assumed best treatment based on evidence, all of which cause medical decisions to be viewed as obstacles. Discrepancies in therapeutic planning, such as unsuitable practices requested by parents, defensive practices for “malignant patients and their family members,” or even malpractice delivered by medical personnel with excessive workloads, were identified by the medical students.

### Theme 5: Development of medical professionalism

**Fig. 5:**
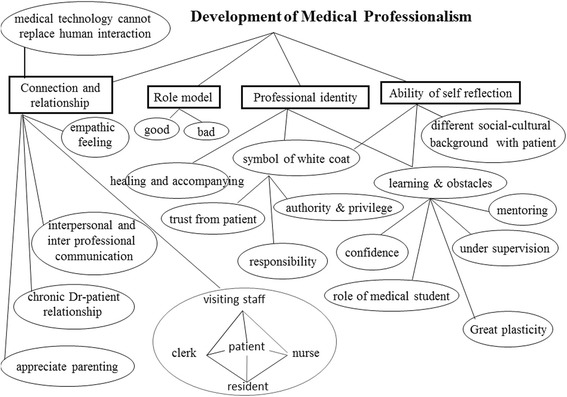
Conceptual map of medical-professional-competence-related psychosocial issues

See Fig. 5. The medical students demonstrated their ability to reflect on their learning processes and roles in clinical encounters. For example, some students admitted to facing learning difficulties or lacking in confidence, even under supervision. In addition, they identified the interactive and complex nature of relationships and interactions with others, noting that most medical technology that cannot replace human interactions in clinical settings. The students also described meeting both good and bad role models during their mentoring terms in clinical care. They often felt empathy for their patients, and were appreciative when young patients had responsible parents (especially those who were present in the pediatric department), and long lasting doctor–patient relationships. Moreover, the medical students developed awareness about the attitude and evident behaviors of competent medical personnel, the symbolism of the white coat, the authority and privilege of doctors, how a patient’s trust is gained, the role of healing and accompany in clinical care, and the responsibility involved in a doctor’s professional identity. Gaining an awareness of the various sociocultural backgrounds of their patients strengthened the medical students’ concepts of multiculturalism and the appropriate relationships between patients and physicians. Finally, reflections and dialogues between group members sensitized the students, and helped them to construct and deconstruct their professional identity, especially those related to their future role as physicians. Overall, the students developed multicultural worldviews that were significantly informed by an enhanced understanding of patient perspectives and stories behind illnesses.

### Theme 6: Public and medical ecological systems

**Fig. 6:**
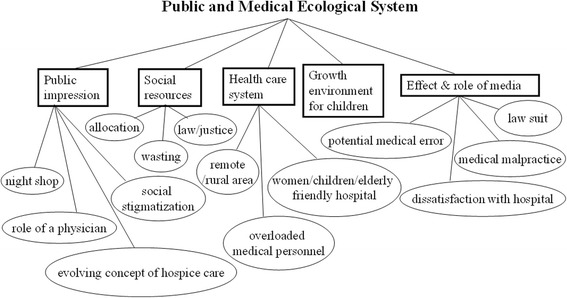
Conceptual map of public-and-medical-ecological-system-related psychosocial issues

See Fig. 6. Public and medical ecological systems refer to social resources, public impressions, the health care system, environments for children that enable growth, and the effect and role of mass media. From the group dialogue, unfavorable growth environments for children, staff shortages, overloaded medical personnel, and an uneven distribution of social resources were noteworthy and repeatedly coded concerns. Moreover, women, children, and elderly-friendly hospitals are embedded in most health care systems. The students noticed that many patients and their family members, and people in general were deeply affected by information delivered through mass media and social media. Many were anxious to follow whatever suggestions they received without consultation with health professionals. Misconceptions, unnecessary medical expenses, and even interference with proper treatment were not unusual under such media influence. Furthermore, the students also perceived medical practices to be viewed with general hostility and noted that they are negatively portrayed by mass media, which is a unique phenomenon. Provocative medical malpractice information, news emphasizing lawsuits or potential medical errors, and inappropriate behaviors from patients in response to medical errors were identified. In addition, social stigmatization, public impressions of the role of physicians, and evolving concepts of hospice care were mentioned by the students as influencing medical practices in clinical settings.

## Discussion

The uniqueness of this study is in its objective to determine psychosocial issues from a structured debriefing process formulated using Gibb’s reflective cycle to address the role of narrative-based medical knowledge in clinical medicine. The medical students in this study initially identified various social and psychological concepts, subsequently attached importance and reacted to such concepts, and related them to their past experiences. After they had reviewed, evaluated, and analyzed the problems with these concepts, they constructed new concepts and formulated action plans to respond to the identified challenges. For example, the students constructed the “meaning of life” concept, and determined that human relationships and connections are central to providing health care after initial contact with patients and listening to stories of their experiences. In addition, the students learned to employ reflective practices during group discussions and demonstrated their ability to reflect their complex and uncertain role in clinical settings and present it as a hidden curriculum from which to learn more about their professional identity. The students were aware of the meaning of life and determined that human relationships and connections are integral in providing health care. The students clearly identified the existence of not only biological events, but also psychological and social factors, that relate to human health.

The main psychosocial issues noted by the students were the extensive connective communication processes in clinical practices, patients’ concerns and their unique perspectives, the role and function of a family, ethical dilemmas in medical encounters, competent actions among medical professionals, and socioeconomic phenomena observed through the form and content of patient stories analyzed as textual units. Such ethical dilemmas could be used as focal points for further discussion to generate more thorough and detailed reasoning and informed judgments. In addition, medical teachers could develop curriculums and learning materials that address these topics based on the structure presented in this study. Medical teachers and students should be able to relate to and draw examples from the stories or information in this study through reflective practice. Identifying these problems enables the students to systematically consider many relevant factors of social and behavioral responses, such as patients’ choices of multidimensional treatment strategies and whether more comprehensive medical care should be offered. Active handling of psychosocial issues should help medical students recognize the roles of other professions, such as those related to psychiatry, family therapy, and social work. A strong inter-professional collaborative team should also be established to deliver holistic medical care. The importance or advantages of interprofessional team work in health services was well reviewed by Reeves, Fletcher, Barr, et al. [16]. Moreover, our finding echoed the importance of facilitating students to learn about being interprofessional in current or future practice [16].

Public and medical ecological systems yielded the largest thematic map, connoting to one of the six core competencies of the Accreditation Council for Graduate Medical Education (ACGME) (i.e., system-based practice, SBP). The focus of this map is to understand each interdependency in a system or series of systems and potential changes for improving health care that can be made and measured in the system [17]. Regardless of national health care and supportive systems, networks based on available regional resources are always present. For example, in this study, the identified living and growing environments for children do not seem suitable from the perspective of the medical students. This remains a crucial concern that influences medical practices, national youth welfare strategies, and police activity, yielding continual calls for reform.

From the students’ perspectives, hostility and negativity toward medical practices was generally portrayed by mass media, representing a unique phenomenon. In particular, provocative medical malpractice information, news emphasizing lawsuits or potential medical errors, and inappropriate behaviors from patients in response to medical errors were identified [18, 19].

The effect and role of social media in the digital world and era of wireless Internet exert positive and negative impacts on health care systems. Mass media and social media have an increasing influence on patients’ treatment-seeking behaviors. Many people seek medical information from resources such as doctors on television programs, medical websites and blogs, newspaper articles written by doctors, and self-diagnosis software. People could misuse the information they receive as it may encourage them to treat themselves, which poses profound challenges to professionalism in medicine.

Meanwhile, these media sources also compel doctors and medical delivery systems to promote their messages through such pipelines. Sufficient and formal medical information and health education, as well as clarification with patients, are necessary with regard to this dilemma. It is clear that the generation of strong networks and secure interactions among physicians, social media, and patients requires considerable attention, especially among medical educators [20, 21].

Medical practitioners should directly face the right to and expectations of health care among patients worldwide. Next-generation physicians should be trained to competently interact and communicate using social media, handle the increase in complaints against doctors, and balance the higher indirect costs of defensive medicine. In addition, the question of how to reduce the discrepancy between the current state of medical science and the capacities of individual doctors and health care authorities through effective use of social media must be addressed [20–22].

This study shows that the societal role of a physician not only involves horizontal networking with other health professionals and health care systems, but is also framed by sociocultural structures and available societal resources. With such a prestigious and privileged role in society, while simultaneously confronting high expectations from patients and the public, medical professionals should have a deeper understanding of psychosocial issues that affect illnesses and treatments, such as poverty, child welfare, community health, and hospitals that are friendly to various demographic populations. Furthermore, medical professionals must act on such understanding to advocate public awareness and policy change, or system reform. Medical professionals and professional groups can apply multiple roles and methods to increase public awareness of medical concerns and cooperate with policy makers to work toward meaningful change; of course, such changes must start from medical education that values the societal role of doctors.

Based on the problems in medical management highlighted by the BPS model and the words of Rita Charon “The sick need people who can understand their diseases: treat their medical problems, and accompany them through their illnesses [23], medicine should be practiced with consideration for science-based medical knowledge and with an understanding of the highly complex psychosocial situations surrounding patients and their family members.” In other words, medical treatment should be structured around patient narratives, and psychosocial issues and the patient’s illness should be treated with equal weight in clinical settings in every case. The marriage between the logical, physician-centered, solid, scientific, evidence-based, biological aspects of medical care, and the patient-centered, doctor*–*patient relationship-based*,* soft skilled, highly plastic, psychosocial, spiritual aspects has clinical significance in the contemporary world. To achieve a high degree of significance, patients’ inherent humanity should be considered and emphasized in medical practices and medical education [23].

A broad range of psychosocial issues were identified by students in their pediatric clerkship. Those selected for and reflected upon in this study are of potential value to medical education. How medical narratives and curriculums can feasibly be designed to integrate such diverse and complex issues should be addressed in the future. This study presented only the descriptive and analytical outcomes without considering intervention strategies, and thus cannot demonstrate behavioral changes among the medical students. Creating and designing clinical courses to help medical students develop the ability to reflect on not only biological but psychosocial problems in clinical settings is a worthwhile endeavor. Furthermore, the educational outcomes of such behavioral changes among medical students should be determined in future studies.

## Conclusions

Through reflective group dialogues, various psychosocial issues in clinical settings were emphasized and discussed. This process enabled medical students to reflect on their experiences and reconstruct uncertain clinical situations involving not only physical but complex BPS issues presented by patients and their family members. Addressing biological, psychological, and social perspectives enables humanistic, holistic, and personalized treatment for patients. Therefore, medical teachers should utilize the core themes and subthemes identified in this study to facilitate further in-depth discussion of psychological issues and cultivate professionalism among medical students in accordance with the BPS model.

Clinical care involves an extremely complex, interactive, and ecological network of biological, behavioral, interpersonal, social, and cultural systems. The discovery of psychosocial issues in this study could bring medical students deeper awareness of these issues from the BPS model perspective and allow them to appreciate patients’ psychosocial narratives.

The pursuit of themes or patterns related to psychosocial issues can be taught to medical students through reflective writing and small group discussion. Qualitative thematic analysis plays a role in identifying, analyzing, and interpreting such issues and illustrates the effectiveness of learning. In summary, a systematic clinical teaching method covering biopsychosocial issues should be developed in the future.

## Abbreviations

ACGME: Accreditation Council for Graduate Medical Education
BPS: Biopsychosocial
SBP: System-based practice
