# Correction to: Psychosocial issues discovered through reflective group dialogue between medical students

**DOI:** 10.1186/s12909-018-1185-3

**Published:** 2018-04-30

**Authors:** Shao-Yin Chu, Chi-Wei Lin, Meei-Ju Lin, Chin-Chen Wen

**Affiliations:** 1 Department of Pediatrics, Buddhist Tzu Chi General Hospital, Hualien, Taiwan; 2 Department of Medicine, College of Medicine, Tzu Chi University, Hualien, Taiwan; 3 Department of Counseling and Clinical Psychology, National Dong Hwa University, Hualien, Taiwan; 4 Department of Human Development and Psychology, Tzu Chi University, Hualien, Taiwan; 5 Department of Counseling and Clinical Psychology, National Dong Hwa University, Hualien, 97401 Taiwan, Republic of China

## Correction

Following publication of the original article [1], the authors reported that they had had mistakenly put the wrong IRB number in the last paragraph of the *Methods* section (as IRB NO.: IRB101–80 instead of IRB102–20 as given in the *Ethics approval and consent to participate in Declarations*). The corrected last paragraph on the Method section should read as follows:

“Ethical approval was obtained from the institutional review board at Buddhist Tzu Chi General Hospital (IRB102-20). Informed consent was received from all participating medical students.”

Furthermore, they found that the name of the first author (Shao-Yin Chu) was not listed in both sets of *Author Comments* (21 Nov 2017, 18 Dec 2017) in their paper’s *Open Peer Review Reports* (https://bmcmededuc.biomedcentral.com/articles/10.1186/s12909-017-1114-x/open-peer-review), where the correspondence author’s name (Chi-Wei Lin) was listed twice.
